# Do Children Who Experience Regret Make Better Decisions? A Developmental Study of the Behavioral Consequences of Regret

**DOI:** 10.1111/cdev.12253

**Published:** 2014-04-29

**Authors:** Eimear O’Connor, Teresa McCormack, Aidan Feeney

**Affiliations:** Queen’s University Belfast

## Abstract

Although regret is assumed to facilitate good decision making, there is little research directly addressing this assumption. Four experiments (*N* = 326) examined the relation between children's ability to experience regret and the quality of their subsequent decision making. In Experiment 1 regret and adaptive decision making showed the same developmental profile, with both first appearing at about 7 years. In Experiments 2a and 2b, children aged 6–7 who experienced regret decided adaptively more often than children who did not experience regret, and this held even when controlling for age and verbal ability. Experiment 3 ruled out a memory-based interpretation of these findings. These findings suggest that the experience of regret facilitates children's ability to learn rapidly from bad outcomes.

Sometimes our choices do not lead to the best possible outcome and to behave adaptively we need to learn to choose differently. For example, when our alarm clock goes off in the morning, we may choose to press the snooze button and thus miss our train to work. To avoid this undesirable outcome, we need to behave differently the next time the alarm goes off. This is an example of what we will term adaptive choice switching: behavior that involves making a different decision when faced with the same or a similar choice again. An intuitively plausible account of how learning occurs in this sort of situation is that the initial choice and its ensuing relatively bad outcome, by virtue of a comparison with an alternative counterfactual possibility, lead to an experience of regret, and this leads to adaptive choice switching (see Zeelenberg & Pieters, 2007). The claim that regret may play a key role in underpinning decision making is particularly interesting in a developmental context, because, as we will discuss, regret is generally considered a complex emotion that emerges relatively late in development. These considerations suggest a simple developmental hypothesis: that the emergence of regret facilitates better decision making in children. Despite a considerable amount of interest in the processes that may underpin improvements in children's decision making, this hypothesis has not yet been tested experimentally. This study reports a series of experiments designed to address this issue.

## Regret and Decision Making

Regret is the negative emotion that occurs when the outcome of a decision is compared unfavorably with an alternative, counterfactual, outcome that could have obtained had a different decision been made (Connolly & Zeelenberg, 2002; Kahneman & Miller, 1986). There has been an explosion of research on regret in neuroeconomics, behavioral decision making, and social psychology. Within this literature, a distinction is made between the effects on decision making of experienced versus anticipated regret (Zeelenberg & Pieters, 2007), with the former referring to the subsequent effects on decision making of experiencing regret, having already chosen poorly, and the latter referring to the effects on one's current decision making of attempting to anticipate and thus minimize future regret. The literature has predominantly been concerned with the anticipation of regret, with economic models of decision making postulating that people weigh up the potential regret that may result from different choices (Bell, 1982; Loomes & Sugden, 1982). Studies have shown that people are regret averse: When choosing they avoid outcomes that they anticipate they will regret (e.g., Josephs, Larrick, Steele, & Nisbett, 1992; Zeelenberg, Beattie, van der Pligt, & de Vries, 1996). There have also been some investigations of the role of experienced regret on adaptive choice, albeit fewer than of anticipated regret. For example, it is known that experienced regrets have consequences for behavior in consumer choice and interpersonal economic contexts such as negotiation and bidding (Ku, 2008; Zeelenberg & Beattie, 1997; Zeelenberg & Pieters, 1999).

As we have said, the well-known formal models of the role of regret have focused on anticipated regret (Bell, 1982; Loomes & Sugden, 1982); accounts of experienced regret are less detailed. Zeelenberg and Pieters (2007) provide the most extensive discussion of this issue, arguing that the primary and distinctive function of experienced regret is to enable people to switch choices when faced with the same decision again. The means by which this occurs is not described in detail, but they claim that experienced regret not only helps us to remember our mistakes and missed opportunities and motivates us to engage in reparative action; by means of mental undoing it also prepares us to behave more appropriately when we are confronted with similar choices in the future (p. 13).

Thus, the process of experiencing regret leads to the evaluation and coding of a choice as a poor one and makes salient alternative courses of action. An alternative analysis is provided by Baumeister, Vohs, DeWall, and Zhang (2007), who recognize a role for experienced emotions in decision making, but suggest that the primary means by which such emotions affect choices is by leading the individual to anticipate negative emotions in the future and thus avoid repeating choices that would lead to such emotions. Although this analysis does not imply that we should drop the distinction between experienced and anticipated regret, it suggests that experienced regret affects subsequent decision making primarily by leading to anticipated regret.

Thus, we can distinguish between at least two ways in which experienced regret may affect decision making. It could be that the experience of regret primes us to anticipate regret in subsequent decisions, resulting in the rejection of a previously selected option due to regret aversion. This is somewhat different from remembering that a particular choice was a poor one (on the basis of the negative emotional experience it yielded) and so, when faced with the same choice again, deciding to avoid the previously chosen option. Neuropsychological studies support the first of these alternatives, providing evidence that the same neuronal circuitry responsible for the experience of regret also underlies the anticipation of regret when a choice is in prospect (Coricelli et al., 2005). While the same areas of the brain may underpin both experienced and anticipated regret, it remains possible that experiencing regret can result in adaptive behavior, independent of the ability to anticipate regret. As we shall see, a developmental study of the effect of regret on decision making is important not just because it may shed light on the degree to which experienced regret is important for adaptive choice switching, but because it may also establish whether experienced regret, independent of anticipated regret, can play a fundamental role in enhancing decision making.

## The Development of Regret

Because regret is a counterfactual emotion (see Kahneman & Miller, 1986) that involves comparing what is to what might have been, the ability to experience regret must develop after the ability to think counterfactually. Empirical studies suggest that counterfactual thought emerges as young as 3–4 years (Harris, German, & Mills, 1996; Riggs, Peterson, Robinson, & Mitchell, 1998), with others arguing that genuine counterfactual thought may develop some years later (see contributions to Hoerl, McCormack, & Beck, 2011, for discussion). The first attempt to experimentally examine when children directly experience regret was described by Amsel and Smalley (2000). In their task, children selected one of two face-down cards and won a small prize if their selected card was of a higher value than that of the experimenter. Once the selected card was revealed, children rated their feelings about their card choice. Children were then shown the card they had not chosen (the counterfactual alternative) and rated their feelings again. Children were classified as experiencing regret if they rated themselves as less happy about their card choice upon seeing that the alternative card would have yielded a win. Amsel and Smalley found that 4- to 5-year-olds did not indicate experiencing regret under these circumstances.

Weisberg and Beck (2010) systematically explored when the ability to experience regret emerges. In their study, children chose between two boxes. The nonchosen box in the regret condition always contained a better prize than the chosen box. Children rated their feelings about their choice before and after they were shown the alternative prize in the nonchosen box. Children were classified as experiencing regret if they reported feeling sadder following the revelation of the nonchosen prize in the regret trial, and in some conditions they did so as early as 5 years. O'Connor, McCormack, and Feeney (2012) modified Weisberg and Beck's task by adding a baseline control condition in which the prize in the nonchosen box was identical to that in the chosen box. They argued that children should be classified as experiencing regret if they report feeling sadder only in the regret condition. Using this criterion, they first found evidence of regret at 6 years (see also Burns, Riggs, & Beck, 2012). Thus, the small number of studies that have been conducted in this area suggest that children first become capable of experiencing regret sometime between 5 and 7 years (although for an exception that places this development much later, see Rafetseder & Perner, 2012). Moreover, the findings indicate that in samples from this age range, we would expect to find some children who are capable of experiencing regret, and some who do not yet report (and so presumably do not experience) this counterfactual emotion. The issue addressed in the current experiments is whether such children also differ in terms of their decision-making abilities.

Although children develop the ability to experience regret between 5 and 7 years of age, they do not appear to be able to anticipate regret until later than this (see also Amsel, Bowden, Cottrell, & Sullivan, 2005). Guttentag and Ferrell (2008) report a study in which participants were shown three boxes, one containing no prize, one containing a medium prize, and one containing a large prize. Participants removed one box, and were then asked to choose one of the remaining two boxes. All participants won a medium prize and were asked what they hoped was in the remaining unopened box. Only by age 9 or 10 years did children (44%) begin to indicate that they hoped there was no prize in the unopened box. Younger children did not anticipate the emotional effects of discovering the big prize in the unopened box, and so they rarely indicated that they hoped it contained no prize. We replicated Guttentag and Ferrell's (2008) findings (McCormack & Feeney, 2014), and have also separately shown that 6- to 7-year-olds who are capable of experiencing regret cannot accurately predict how they would feel if an as-yet-unknown outcome turns out to be better than one that resulted from their choice.

Although the ability to experience regret develops between the ages of 5 and 7 years, the ability to anticipate regret seems to develop somewhat later. Thus, a study of the relation between experienced regret and decision making in children aged between 5 and 7 years has the potential not only to investigate hypotheses about the centrality of experienced regret to the development of adaptive decision making but also to test whether the experience of regret can result in adaptive decision making at an age at which it seems likely that children are incapable of anticipating regret.

## The Current Experiments

The obvious developmental hypothesis that emerges from a consideration of the literature on experienced regret and decision making is that when children begin to experience regret, their subsequent decision making will improve. This hypothesis rests on the assumption that experiencing regret can affect decision making by a process that does not involve anticipating regret. Given Zeelenberg and Pieters's (2007) claim about the basic role of experienced regret in choice switching, the prediction is that, following a nonoptimal decision, children who experience regret will be more likely to make a better decision when faced with the same choice again than those who do not. That is, when the ability to experience regret emerges developmentally, it should be accompanied by improvements in what we have termed *adaptive choice switching*. No research studies have yet addressed this issue. Two studies, those of Burnett, Bault, Coricelli, and Blakemore (2010) and Habib et al. (2012), have examined reported regret alongside decision making in older children, but they were not designed to test this specific hypothesis. Their studies examined more complex decision making in a gambling task in which participants decided between two gambles that varied in associated risk (Camille et al., 2004). Habib et al. found developmental increases between 11 years and adulthood in reported regret (although see Burnett et al., 2010). Neither of these studies found a link between regret and decision making. However, the youngest participants were some years older than the age at which regret is thought to emerge developmentally (O'Connor et al., 2012; Weisberg & Beck, 2010) and these studies did not assess adaptive choice switching per se. Moreover, because gambling paradigms involve many trials, they make it almost inevitable that any regret effects will be due to anticipated rather than experienced regret. On tasks with multiple trials, it may be difficult to remember discrete experiences or to gauge the relation between a current gamble and previous gambles.

In our experiments, we used a simple task to examine whether there is a developmental relation between regret and decision making, in which children made a decision on the first day, discovered the outcome of this decision, and then had to make the same decision again the following day. In this sense, our task resembles an experimental version of the sort of everyday situation we began our introduction with (the alarm clock example), in which behaving adaptively involves making a different choice when faced with the same decision again. The task was designed to examine both whether children experienced regret when they discovered the consequences of their initial choice and whether they then adaptively switched choices when faced with the same situation again. As in O'Connor et al.'s (2012) study, children chose between a pair of boxes in regret and baseline conditions. By comparing children's happiness ratings before and after the outcomes of both choices were known, we measured whether they experienced regret. The next day, children were presented with the same choices again. The payoff structure on the 2nd day means that to maximize their outcome, children should switch choice in the regret condition only. We compared the rate of adaptive choice switching among children who experienced regret on Day 1 with those who did not, with our prediction being that children who experience regret will show higher rates of adaptive switching on Day 2.

## Experiment 1

### Method

#### Participants

Twenty-six 5-year-olds (13 females, *M *=* *62.3 months, range = 60–66 months), twenty-eight 7-year-olds (14 females, *M *=* *85.5 months, range = 83–89 months), and twenty-four 9-year-olds (15 females, *M *=* *110.1 months, range = 108–113 months) participated. In this and other experiments, children were predominately from lower- to middle-class backgrounds and of Caucasian origin.

#### Apparatus

The stimuli were four differently colored boxes (25 × 14 × 21 cm), each with a distinctive picture on its lid. Two colored boxes were used for the regret condition and two for the baseline condition. The baseline colored boxes each contained a smaller silver box with one plastic token inside. The regret colored boxes each contained two smaller silver boxes. One of these silver boxes contained one token and the other contained 10 tokens. The silver boxes were designed so that they could be distinguished by touch alone, with one box having a bumpy surface.

A 5-point affective response scale was used, with each response point illustrated by one of five cartoon faces varying in emotional expression from very happy on the left to very sad on the right. Children indicated their affective response to an outcome by placing a three-pronged arrow pointing at the appropriate face. This arrow had one prong pointing upward, and a leftward- and a rightward-pointing prong.

#### Procedure

All testing took place in a quiet room of the participant's school. On Day 1, participants were invited to play a game in which they could win tokens that would be exchanged for stickers once the game was over. First, the scale was carefully explained to participants in four practice trials using two puppets. In each of the practice trials participants saw one puppet receiving a gift and were asked to indicate on the scale how they thought the puppet would feel. Next they saw the puppet receive an additional gift or have part of their initial gift taken away. Participants were asked to indicate, using the three-pronged arrow, whether the puppet would feel happier (leftward prong), sadder (rightward prong), or the same (upward-pointing prong). Further details of this training procedure are provided in O'Connor et al. (2012). The experiment did not commence until participants had given the correct answers on all four practice trials and participants who gave wrong answers were corrected.

Once the practice trials had been completed, participants were shown the baseline condition boxes and were told that they contained tokens. The baseline condition was always administered first, because previous research suggested that this maximizes the likelihood of experiencing regret in the regret condition (O'Connor et al., 2012). Children were given a single trial in each condition to avoid the possibility that learned expectations about box contents might affect emotional ratings. In the baseline condition, children selected one colored box and the smaller silver box was removed from the chosen box and opened to reveal one token. Once children had expressed their affective response to the outcome, the experimenter showed them the prize that they could have won had they chosen the other box, which was also one token. Using the three-pronged arrow, participants indicated whether they felt happier, sadder, or the same as before. Next, the experimenter put the tokens back inside the silver boxes, which were placed inside the baseline boxes, and wrote the child's name on their chosen box, explaining that the same game would be played again the following day with the same prizes in the same boxes. The procedure for the regret condition was identical except that regardless of which colored box was chosen, the experimenter always retrieved the silver box containing one token and the counterfactual prize retrieved from the nonchosen box was always 10 tokens. The experimenter counted the 10 tokens from the nonchosen box in front of each participant. The experimenter retrieved the appropriate silver box from the regret boxes by identifying it by touch, and children were unaware that there was more than one silver box inside each colored box. After children had completed both trials they received two stickers.

Participants were called back the next day. First, they were reminded that their name had been written on the boxes they had previously chosen and were told that the boxes contained the same prizes as they had before. Participants were given the same choices in the same order as on the previous day. Prior to each choice, the experimenter gave participants a token and showed them both colored boxes, making it clear which box had been chosen the day before. It was explained to participants that they could exchange the token they had just received in order to switch their choice, or they could choose the box they had previously chosen for free. The prize in the baseline condition was one token regardless of box choice and one token in the regret condition if participants stuck with their original choice, but 10 tokens if they switched.

We included a baseline condition to control for the possibility that children might switch in the regret condition for reasons unrelated to the adaptivity of switching. The observation that children switched in both conditions would suggest that switching was simply their preferred response, most likely due to a general preference for novelty. Note that this procedure included a cost of one token for switching on Day 2. This was because pilot work established that switching was indeed many children's preferred response, regardless of condition. We hoped that the introduction of a switching cost would lead participants to switch only when it was profitable to do so.

We ran a posttest to ensure that the youngest participants were capable of the simple arithmetic required to calculate the potential costs and benefits of switching. Participants saw a puppet who was said to have one present and to be about to get another. Another puppet was said to have one present which she was due to lose but would then get 10 more. Participants were asked which puppet would then have most presents. All participants gave the correct answer.

### Results

In Table1 we report the numbers of participants who felt happier, sadder, and the same in each condition once they saw the counterfactual prize. Few of the youngest children reported feeling sadder in the regret condition but that almost all of the older children did. Participants who reported feeling sadder in the regret condition only were coded as having experienced regret.

**Table 1:** Numbers of Children Giving Each Emotional Response on Day 1 After the Counterfactual Prize Was Revealed in Each Condition in Experiment 1, Broken Down by Age Group and Whether Participants Were Classified as Having Experienced Regret

Age group	Experience regret	Baseline condition			Regret condition		
		Happier	Sadder	No change	Happier	Sadder	No change
5	Yes (*N *=* *6)	2	0	4	0	6	0
	No (*N *=* *20)	1	3	16	6	0	14
7	Yes (*N *=* *19)	4	0	15	0	19	0
	No (*N *=* *9)	2	0	7	0	0	9
9	Yes (*N *=* *23)	6	0	17	0	23	0
	No (*N *=* *1)	0	0	1	0	0	1

In Table2 we present switching data from Day 2 for each condition broken down by age group and experienced regret. Many of the younger children seemed to prefer to switch on Day 2 regardless of condition, while very few of the oldest participants switched in the baseline condition. Subsequent analysis controlled for nonadaptive choice switching in the baseline condition. Participants who had switched in the regret condition only were coded as adaptive switchers. All other switching patterns were coded as nonadaptive. Overall, there was a significant association between whether participants experienced regret on Day 1 and their decision making on Day 2, χ^2^(1, *N *=* *78) = 20.05, *p *<* *.001, Φ_C_ = .51. Fisher exact probability tests revealed that the association did not reach significance for any of the individual age groups, primarily because within each age group the large majority of children either experienced or did not experience regret (all *p*s > .2). Nonetheless, subsequent analysis revealed that rates of adaptive switching in some groups differed from those expected by chance. Because there were three possible nonadaptive switching patterns and only one adaptive pattern, the probability of switching adaptively due to chance was .25. Binomial tests revealed that rates of adaptive switching among 7- and 9-year-olds who experienced regret were highly unlikely to be due to chance (in both cases, *p *<* *.001). Among 5- and 7-year-old participants who did not experience regret, rates of adaptive switching did not differ from those expected by chance.

**Table 2:** Numbers of Children Showing Each Pattern of Choices on Day 2 in Experiment 1 Broken Down by Age and Whether Participants Experienced Regret in the Regret Condition

Age (years)	Experience regret	Baseline condition		Regret condition		Adaptive switching	
		Switch	No switch	Switch	No switch	Yes	No
5	Yes (*N *=* *6)	5	1	5	1	0	6
	No (*N *=* *20)	14	6	12	8	1	19
7	Yes (*N *=* *19)	4	15	17	2	14	5
	No (*N *=* *9)	4	5	7	2	4	5
9	Yes (*N *=* *23)	4	19	22	1	19	4
	No (*N *=* *1)	0	1	0	1	0	1

Note. The two leftmost data columns show the numbers of children who switched choices in the baseline condition. The middle data columns show the equivalent numbers in the regret condition. The rightmost data columns show the numbers of children who switched adaptively (i.e., switched in the regret but not baseline condition).

### Discussion

Adaptive choice switching in this task appears to emerge at the same time as the ability to experience regret. Five-year-old participants rarely experienced regret upon learning that a counterfactual prize was better than their actual prize, whereas almost all 9-year-olds experienced regret. Similarly, only one 5-year-old switched choices adaptively, whereas nearly all 9-year-olds did. A substantial proportion of 7-year-olds experienced regret; adaptive choice switching was also observed in the majority of children this age group. Furthermore, regret and adaptive choice switching appear to be associated. Participants who experienced regret were significantly more likely to switch adaptively than those who did not. These results provide some initial support for the suggestion that the development of regret allows children to learn from previous decisions in order to adaptively switch their choices.

In our paradigm, adaptive choice switching was defined not just as switching choices in Day 2 in the regret condition, but, in addition, not switching on the baseline condition. In the regret condition, 5-year-olds' performance was not very dissimilar to that of the older groups: 65% of the younger children choose to switch box on Day 2. However, unlike in the older groups, a similar percentage of 5-year-olds also switched choice in the baseline condition. This finding indicates that switching choices in the regret condition was not always a result of good decision making, because it suggests that some children may have had a general preference for switching. Therefore, it is important to examine whether children selectively switch their choices only when it is advantageous to do so, and how this relates to the experience of regret.

The results of this experiment on its own do not provide convincing evidence that children who experience regret are better decision makers. Because of the wide age range employed, there were large subgroups of children, defined by age, who either did or did not experience regret. Thus, whether or not a participant was capable of experiencing regret was confounded with age. As many abilities develop with age, it is difficult to make causal claims about the link between the ability to experience regret and the tendency to switch adaptively based on a sample with such an age range. Our aims in the next experiments were to replicate the association between experienced regret and choice switching in 6- and 7-year-old children, controlling for age. We chose this age group because children in the 7-year-old sample in Experiment 1 varied in the ability to experience regret.

## Experiment 2a

### Method

#### Participants

Seventy-one 6- to 7-year-olds (36 females, *M *= 80.6 months, range = 72–92 months) participated.

#### Apparatus and Procedure

These were identical to those used in Experiment 1.

### Results and Discussion

One child reported feeling sadder after the counterfactual prize was revealed in both conditions. Due to the difficulty in interpreting this pattern of performance, the data from this child were excluded from the analyses. The numbers of participants who felt happier, sadder, and the same after seeing the counterfactual prize in each condition are reported in Table3. Rates of choice switching in each condition on Day 2, broken down by whether participants experienced regret, are presented in Table4. Slightly more participants experienced regret than participants did not experience the emotion. In the regret condition, switching appears to be strongly associated with the experience of regret. The majority of participants did not switch in the baseline condition, although there was a sizable minority that did switch, regardless of whether they had experienced regret. Once again, we controlled for nonadaptive choice switching in the baseline condition. A chi-square test revealed a significant association between the experience of regret on Day 1 and adaptive choice switching on Day 2, χ^2^(1, *N *=* *70) = 9.61, *p *<* *.005 Φ_C_ = .37. To ensure that the association between regret and adaptive choice switching is not confounded with age, we carried out a binary logistic regression with adaptive choice switching on Day 2 as the outcome variable, using age in months and whether participants experienced regret on Day 1 as the predictor variables. The results of this analysis are presented in Table5. While regret on Day 1 was a significant predictor of adaptive choice switching on Day 2, age in months was not. Thus, within this sample an increase in age did not lead to more adaptive choice switching on Day 2 and, more important, controlling for age, the association between regret and adaptive choice switching was statistically significant.

**Table 3:** Numbers of Children Giving Each Emotional Response on Day 1 After the Counterfactual Prize Was Revealed in Each Condition in Experiments 2-3, Broken Down by Whether Participants Were Classified as Having Experienced Regret

Experience regret	Baseline condition			Regret condition		
	Happier	Sadder	No change	Happier	Sadder	No change
Experiment 2a						
Yes (*N *=* *38)	9	0	29	0	38	0
No (*N *=* *32)	7	1	24	7	0	25
Experiment 2b						
Yes (*N *=* *61)	20	0	41	0	61	0
No (*N *=* *52)	14	3	35	6	0	46
Experiment 3						
Yes (*N *=* *36)	12	0	24	0	36	0
No (*N *=* *24)	7	0	17	5	0	19

**Table 4:** Numbers of Children Showing Each Pattern of Choices on Day 2 in Experiments Experiment 2a and Experiment 2b Broken Down by Whether Participants Experienced Regret in the Regret Condition

Experience regret	Baseline condition		Regret condition		Adaptive switching	
	Switch	No switch	Switch	No switch	Yes	No
Experiment 2a						
Yes (*N *=* *38)	11	27	37	1	26	12
No (*N *=* *32)	17	15	18	14	10	22
Experiment 2b						
Yes (*N *=* *61)	19	42	53	8	38	23
No (*N *=* *52)	21	31	36	16	18	34

Note. The two leftmost data columns show the numbers of children switching choices in the baseline condition. The middle data columns show equivalent numbers for the regret condition. The rightmost data columns show the numbers of children who switched adaptively (i.e., switched in the regret but not baseline condition).

**Table 5:** Results of the Binary Logistic Regression Analyses on Adaptive Choice Switching From Experiments Experiment 2a and Experiment 2b

	95% CI for Exp b			
	*B* (*SE*)	Lower	Exp b	Upper
Experiment 2a				
Regret on Day 1	1.60* (0.52)	1.78	4.96	13.87
Age in months	0.03 (0.04)	0.96	1.03	1.12
Constant	−3.33 (3.25)		0.04	
Experiment 3				
Regret on Day 1	1.12* (0.42)	1.36	3.08	6.96
Age in months	−0.02 (0.03)	0.92	0.98	1.04
Raw BPVS scores	0.01 (0.02)	0.98	1.01	1.04
Constant	0.59 (2.47)		1.8	

Note. For Experiment 2a, *R*^2^ = .12 (Hosmer & Lemeshow), .14 (Cox & Snell), .19 (Nagelkerke); model χ^2^(2) = 10.48, *p* < .006. For Experiment 2b, *R*^2^ = .12 (Hosmer & Lemeshow), .14 (Cox & Snell), .19 (Nagelkerke); model χ^2^(2) = 10.48, *p* < .006. BPVS = British Picture Vocabulary Scale.

* *p* < .003.

In order to control for a tendency to switch on Day 2, we defined adaptive decision making with reference to both the regret and baseline conditions. It is important, although, to show that the association with the ability to experience regret holds in the regret condition, independently of performance in the baseline condition. Examination of Table4 suggests that these conditions differ in the nature of their association with whether regret is experienced. That is, participants who experienced regret on Day 1 were more likely to switch in the regret condition than were participants who did not experience regret, χ^2^(1, *N *=* *70) = 17.44, *p *<* *.001, Φ_C_ = .50, whereas participants who did not experience regret were somewhat more likely to switch in the baseline condition than did participants who experienced regret, χ^2^(1, *N *=* *70) = 4.23, *p *<* *.05, Φ_C_ = .25. These results indicate that the experience of regret is associated with adaptive switching in the regret condition independent of performance in the baseline condition.

These results support our hypothesis that for adaptive choice switching to be reliably observed in our task, children must be capable of experiencing regret, and in this experiment age was not related to whether participants were capable of regret following a bad choice outcome. In addition, because it seems unlikely that children of the age range used in this study are able to anticipate regret (Amsel et al., 2005; Guttentag & Ferrell, 2008), these results also support the hypothesis that the experience of regret is associated with adaptive decision making, independent of the anticipation of regret.

## Experiment 2b

Experiment 2b was identical to Experiment 2a, but we also controlled for children's verbal ability.

### Method

#### Participants

One hundred and seventeen 6- to 7-year-olds (62 females, 55 males, *M *=* *82.4 months, range = 72–95 months) participated.

#### Apparatus

The materials used were the same as the first two experiments. Each participant completed the British Picture Vocabulary Scale II (BPVS II; Dunn, Dunn, Whetton, & Burley, 1997), which is a standardized measure of receptive vocabulary.

#### Procedure

At the start of the testing session on Day 1 the BPVS was administered to each participant; the remaining procedures for Day 1 and Day 2 were identical to those used Experiments Experiment 1 and Experiment 2a.

### Results and Discussion

Table2 shows the Day 1 responses for regret and baseline conditions; as in Experiment 2a, the majority of children reported feeling sadder in the regret condition but the same in the baseline condition. The data from four children who reported feeling sadder after the counterfactual prize revealed in both conditions were excluded from analyses. For the remaining 113 participants, rates of choice switching on Day 2 in each condition, and rates of adaptive choice switching, both broken down by whether participants experienced regret, are presented in Table4. More participants experienced regret on Day 1 than did not experience regret, but, as was the case in Experiments Experiment 1 and Experiment 2a, the experience of regret appears to be associated with adaptive choice switching on Day 2. A chi-square test showed this association between regret on Day 1 and choice switching on Day 2 to be significant, χ^2^(1, *N *=* *113) = 8.60, *p *<* *.005, Φ_C_ = .28. In this experiment, the association between experienced regret and switching in the regret condition was statistically significant, χ^2^(1, *N *=* *113) = 5.23, *p *<* *.03, Φ_C_ = .22, but the association between experienced regret and switching in the baseline condition was not, χ^2^(1) = 1.05, *p *>* *.1. Thus, the ability to experience regret is associated with adaptive choice switching whether or not one controls for a general tendency to switch.

Participants' mean standardized score on the BPVS was 100.43 (*SD* = 10.86). We carried out a binary logistic regression with adaptive choice switching on Day 2 as the outcome variable and raw BPVS scores, age in months, and whether regret was experienced on Day 1 as predictor variables. The results of this analysis are presented in Table5 where it may be seen that regret on Day 1 was the only significant predictor of adaptive choice switching on Day 2. Because the BPVS is often used as a proxy for general intelligence, our finding that adaptive switching on Day 2 is not associated with BPVS scores could be understood as suggesting that the relation between the ability to experience regret and adaptive decision making is a direct one, and is not driven by effects of intelligence measured separately from age.

## Experiment 3

Thus far our results show that the ability to experience regret is associated with adaptive choice switching. However, there are a number of reasons why such an association may hold. There may be a direct relation between the ability to experience regret and adaptive decision making or the relation may be more indirect (e.g., as a result of a separate mediating variable). That the association holds when BPVS scores and age are separately controlled for is evidence for a direct rather than indirect relation. However, so far we have not provided any evidence regarding the processes that might underpin such a direct relation. One possible process is memory: An inability to experience regret might be associated with poorer recall of Day 1 outcomes, which may lead to poor decision making on Day 2. It may be that experiencing regret facilitates memory for the actual contents of the boxes, in line with suggestions that emotion has an impact on memory accuracy (e.g., Bradley, Greenwald, Petry, & Lang, 1992; Christianson & Loftus, 1987; McGaugh, 2002), and that remembering the contents accurately is necessary for adaptive choice switching in this task. Indeed, as Baumeister et al. (2007) discuss, one of the means by which emotions might affect learning is by facilitating memory for aspects of the situation that resulted in the emotion.

To examine this possibility, in Experiment 3 we asked all participants, before they made their choice on Day 2, to recall the contents of each box. A number of results were possible. If memory differences underlie the association observed in Experiments 1-2b, then we would expect participants who did not experience regret to have poorer memory for the contents of the boxes on Day 2 than participants who did experience regret. Alternatively, all participants may have equally good memory for the box contents when asked explicitly, but perhaps those who experienced regret in our previous experiments were more likely to spontaneously bring those memories to bear on the task on Day 2. If this is correct, all participants may do equally well when probed with memory questions, and because of those questions, they may do equally well on the decision-making task in the regret condition. A third possibility is that participants will not be able to answer the explicit memory questions, but nevertheless, as in previous experiments, regretters will show adaptive choice switching on Day 2. This possibility is interesting, because it would suggest that regret can serve like a reinforcement process, marking a choice as a bad choice, without requiring recollection of why this is the case.

In addition to introducing memory questions, we included a further task to assess children's counterfactual thinking taken from Amsel et al.'s (2014) study, which we labeled the alternative outcome task. This task was similar to our regret task, in that children made a choice between two containers and then received a prize. Children were then shown the counterfactual prize and were asked to rate how they would feel if they had received it. The critical difference between the alternative outcome task and the regret task is that in the latter, children must report on how they feel about their actual choice, whereas in the alternative outcome task, children need only consider how they would feel if they had received a different prize. Amsel et al. found that children were able to report how they would feel if they had received a better prize at an earlier age (4–5 years) than they showed evidence of regret (6 years). As Amsel et al. point out, the experience of regret has two cognitive components. First, it involves the ability to think counterfactually about alternative states of affairs and the emotional consequences of those alternatives. Second, it involves bringing to bear knowledge about those alternatives and their emotional consequences to assessments about the actual outcome that a choice has resulted in. The alternative outcomes task assesses the first of these abilities, whereas only the regret task assesses the second one. If children who fail to show regret in the regret condition also fail in this task, it would suggest that they not just fail to experience regret about their choices but they have more global problems reasoning about counterfactual scenarios. Such problems in counterfactual thinking rather than in experiencing regret per se may lie behind the group differences we have found.

### Method

#### Participants

Sixty 6- and 7-year-olds (29 females, *M *=* *78.8 months, range = 72–90 months) participated.

#### Apparatus and Procedure

With the exception of two changes on Day 2, the basic apparatus and procedure were identical to those used in Experiments 1-2b. The first change on Day 2 involved the inclusion of memory questions. At the start of each condition on Day 2, before the participant chose a colored box, the researcher pointed to one box and asked the child, “Do you remember how many tokens are in this box?” Once the child responded the researcher pointed to the other box and asked the recall question again. The presentation of boxes in each condition was counterbalanced and children received no feedback on their responses. The second change was that an additional counterfactual understanding assessment—the alternative outcome task—was introduced at the end of the decision-making task. In this task, two identical bags containing either one or six coloring pencils were used. The participant was asked to choose one bag for a prize and to rate how they felt about their prize on the 5-point affective scale. The participant was then shown the prize from the unchosen bag and was asked, “How would you have felt if you had chosen this bag instead?” Participants indicated their response in the same manner as they had in the main part of the experiment. Outcomes on this task were not rigged, with the result that some of the children had to make a judgment about how they would feel if they had received the better prize of six pencils (*N *=* *35), and some had to judge how they would feel if they had received the worse prize of one pencil (*N *=* *25).

### Results and Discussion

The majority of participants (87%, *N *=* *52) answered the counterfactual question correctly in the alternative outcome task, indicating that they would have felt happier if they had won six coloring pencils and sadder if they had won one coloring pencil. Four of the eight children who did not give the appropriate response experienced regret and four did not. These results suggest that children who failed to experience regret nevertheless have the basic ability to reason about the emotional consequences of counterfactual scenarios.

The responses given by participants to the Day 2 memory questions are shown in Table6. In the regret condition we coded as correct answers giving the precise number of tokens in each box or indicating that there were “more” or “lots of” tokens in the nonchosen box. Only one participant answered the memory question incorrectly in the regret condition. In the baseline condition, eight participants answered the memory question incorrectly by indicating that there had been one token in the box they chose and two tokens in the other box, or that there had been two tokens in each box. Of these nine participants who gave an incorrect answer, six experienced regret on Day 1 and three did not. Thus, regardless of whether or not participants experienced regret on Day 1, they were able to remember the contents of the boxes when explicitly asked to do so. The analyses that follows includes all 60 children; however excluding children who incorrectly answered either a memory question or the test question in the alternative outcome task did not alter the results.

**Table 6:** Performance on the Memory Questions in Experiment 3, Broken Down by Whether Children Were Classified as Having Experienced Regret on Day 1

Experience regret	Baseline condition			Regret condition		
	1 + 1 token	1 + 2 tokens	2 + 2 tokens	1 + 10 tokens	“Lots”/“More” tokens	1 + 1 tokens
Yes (*N *=* *36)	31	3	2	31	4	1
No (*N *=* *24)	21	3	0	19	5	0

Note. “1 + 2” and “2 + 2” responses in the baseline condition and “1 + 1” responses in the regret condition are inaccurate. “Lots”/“More” responses in the regret condition indicate that children could remember that there were more tokens in the nonchosen box but not the exact number of tokens.

Table3 shows that on Day 1, as in Experiments Experiment 2a and Experiment 2b, the majority of children felt sadder in the regret condition but the same in the baseline condition after the counterfactual prize was revealed. On Day 2, almost all participants (35 of 36 regretters and 21 of 24 nonregretters) switched boxes in the regret condition. These results are strikingly different to earlier results in that the vast majority of participants in this experiment switched in the regret condition regardless of whether they had experienced regret on Day 1. As a result, unlike the findings from Experiments Experiment 2a and Experiment 2b, there was no association found between the experience of regret on Day 1 and switching in the regret condition, Fisher exact probability = .29. However, as was the case in Experiment 2a, a significant association was found between the experience of regret and switching in the baseline condition, χ^2^(1, *N *=* *60) = 7.14, *p *<* *.01 Φ_C_ = .35, such that participants who did not experience regret were more likely to switch than those who experienced regret. This was because only 10 of 36 regretters switched in the baseline condition, but 15 of 24 nonregretters did so.

The results of this experiment rule out memory differences as a potential cause of the association between the experience of regret and adaptive choice switching: Children had very good memory for box contents and their memory was equally good regardless of whether they experienced regret on Day 1. Likewise, the results rule out more basic differences in the ability to think counterfactually as a cause of the association; even 6- to 7-year-olds who fail to experience regret were able to answer a simpler question about the emotional consequences of a counterfactual scenario.

Although this experiment does not establish exactly how the experience of regret has an impact on decision making, we can speculate on this process on the basis of its findings. Once participants answered the memory question, they overwhelmingly switched adaptively in the regret condition, regardless of whether they had experienced regret on Day 1. This finding suggests that the experience of regret has its effects via its consequences for whether participants spontaneously bring their memory of the original choice outcomes to bear on Day 2. Children who experience regret will have made an evaluative comparison about the counterfactual outcome on Day 1 relative to the actual outcome, and thus, when faced with the same situation, seem to spontaneously bring to mind the relative values of different outcomes, which then inform their decision to switch adaptively. By contrast, children who do not experience regret seem to need to be explicitly asked to bring to mind the relative values of the outcomes.

Although for the sake of brevity we have not reported the findings previously, we asked participants in Experiments 1-3 to explain their choices on Day 2 when they decided to switch. We introduce these findings now because they are relevant to the interpretation of the data from Experiment 3. We coded these explanations (see Table7) as the desire to swap (corresponding to something like a novelty preference), the need to choose adaptively (typically involving reference to one of the boxes having the better prize), or to some other unrelated factor (e.g., a preference for one of the pictures on a box). An examination of Table7 reveals that in the regret condition the majority of explanations given by participants who experienced regret in Experiments 1-2b involved adaptive switching. On the other hand, this was only true of participants who did not experience regret when they were asked the memory question in Experiment 3. These explanations provide further evidence that participants who experienced regret explicitly and spontaneously recalled Day 1 decision outcomes when making their choice on Day 2, whereas those who did not experience regret did not do so unless prompted.

**Table 7:** The Number of Children Giving Each Type of Explanation for Changing Box Choice on Day 2 in Experiments 1-3, Broken Down by Whether Participants Experienced Regret on Day 1

Experience regret	Baseline condition		Regret condition		
	Desire to swap	Unrelated	Desire to swap	Adaptive switching	Unrelated
Experiment 1					
Yes (*N *=* *48)	8	5	7	30	7
No (*N *=* *30)	5	13	3	6	10
Experiment 2a					
Yes (*N *=* *38)	6	5	5	28	4
No (*N *=* *32)	11	6	3	10	5
Experiment 2b					
Yes (*N *=* *61)	8	11	4	36	13
No (*N *=* *52)	4	17	5	14	17
Experiment 3					
Yes (*N *=* *36)	3	7	1	30	4
No (*N *=* *24)	8	7	3	15	3

Although we found that regretters and nonregretters behaved similarly in the regret condition on Day 2, these groups differed in the likelihood that they would switch in the baseline condition (a nonadaptive switch; this group difference on baseline condition was also found in Experiment 2a but not in Experiment 2b). This result is puzzling because in Experiment 3 both groups of children successfully recalled that there was one token in each box before making their Day 2 choice, yet the nonregretters were more likely switch boxes. Inspection of Table7 shows that around half of the nonregretters who switched in the baseline condition in Experiment 3 explained their decision in terms of simply wanting a different box, and they differ from the regretters in this respect. It may be that there are some children who are prepared to opt for a slightly worse outcome (one token rather than two tokens) to satisfy their novelty preference; why these children are more likely to be those who did not experience regret is unclear. Alternatively, it could be that these children did not properly evaluate the difference between their choices and did not realize that they would be opting for a worse outcome, even though the mathematics involved was very simple. Arguably, this latter suggestion fits better with the finding that these children were more likely to have been from the nonregretter group—children who did not seem to spontaneously evaluate the difference between better and worse outcomes on Day 1.

## General Discussion

This study is the first to simultaneously examine regret and decision making in young children. The results of Experiment 1 show that the ability to switch a decision adaptively given a previous relatively bad outcome emerges at the same time in development as the ability to experience regret. The results of Experiment 2a show a strong association between the ability to experience regret and adaptive choice switching when controlling for age, and those from Experiment 2b show the same association when also controlling for verbal ability. We interpret these results as suggesting that the experience of regret itself may facilitate this form of adaptive choice switching in children.

The findings of Experiment 3 allow us to speculate about the processes that might underpin the facilitative effect of regret in our task. When explicitly asked to recall the contents of the boxes from Day 1, even children who had not experienced regret were very accurate at remembering what the boxes contained. Moreover, they were then able to adaptively switch their choices in the regret condition and to justify this decision with reference to the difference in contents between the boxes. This contrasted with the performance of this group of children in the previous experiments, in which they were likely to fail to switch choices in the regret condition and even when they did switch often justified their choice in terms of nonadaptive reasons. These findings suggest that all children were highly aware on Day 1 of the prize in the nonchosen box. However, those who experienced regret were much more likely to bring this information to bear when faced with the same choice on Day 2 (Experiments 1-2b). Thus, in our task, previously experienced regret seems to predispose decision makers toward explicit recall of the outcomes associated with particular decisions when those options are reencountered.

Further studies are necessary to establish whether this is the correct interpretation of our findings, and, if so, to what extent such a mechanism might generalize to other tasks. We note, though, that this explanation of our findings suggests a simple way in which experienced regret might impact on decision making without the involvement of anticipatory regret. The general issue of whether it is the experience of or the anticipation of an emotion that primarily has behavioral consequences is a matter of considerable debate (e.g., Baumeister et al., 2007; Loewenstein, Weber, Hsee, & Welch, 2001). As noted in the Introduction, formal models of regret and decision making have focused on how anticipated regret might impact on people's choices and the vast majority of empirical studies with adults examining the effects of regret on decision making have studied the behavioral consequences of anticipated rather than experienced regret (e.g., Mellers, Schwartz, & Ritov, 1999; Simonson, 1992; Zeelenberg et al., 1996). Our results suggest that the experience of regret is associated with adaptive decision making in children who are several years younger than the age by which the ability to anticipate regret is thought to develop (Amsel et al., 2005; Guttentag & Ferrell, 2008). It may be that once anticipatory regret emerges there are additional, and perhaps more widespread, effects on children's decision making; we see this as an important direction for future research (see also Amsel et al., 2005). However, the current results suggest that there is a means by which experienced regret per se can affect behavior, indicating that the emergence of regret may have important developmental consequences.

We have broadly characterized our findings as indicating that once children are capable of experiencing regret, they engage in better decision making. However, what we have found is an association in a specific context between whether or not children report regret and the quality of their subsequent decision making. Framed as a developmental claim, our interpretation of the findings assumes that children who do not report regret in our task are not yet capable of doing so. It remains possible that children we characterized as “nonregretters” may be capable of experiencing regret under different circumstances (e.g., if the counterfactual alternative were particularly attractive), and it is important in future research to widen the scope of paradigms used to assess regret in children. We doubt, though, that the failure of young children to demonstrate regret is merely task dependent. Rather, there are cognitive prerequisites that are required for experiencing regret that some children may not yet have developed. A basic role for cognition is clear if we consider that experiencing regret is only possible if children are able to think counterfactually. However, there appears to be a developmental lag between the emergence of counterfactual thinking and the ability to experience regret (Amsel & Smalley, 2000; Amsel et al., 2014; Beck, Riggs, & Burns, 2011; Perner & Rafetseder, 2011). This suggests that the ability to think counterfactually is necessary but not sufficient for the experience of regret. The findings of Experiment 3 also support this suggestion: Children who did not show evidence of regret were nevertheless able to reason that they would have felt happier if they had been given a better prize (the alternative outcome task). Beck et al. (2011) have argued that regret involves more complex cognitive processes than are typically tapped in tasks assessing children's counterfactual reasoning. In at least some counterfactual reasoning tasks, children are required to hold two possible worlds in mind: the actual world and the counterfactual world (Beck, Robinson, Carroll, & Apperly, 2006; see Perner & Rafetseder, 2011, for discussion). Beck et al. (2011) argue that experiencing regret involves making spontaneous evaluative comparisons between these worlds, and that this requires being able to flexibly switch between representations. Some initial evidence for this suggestion comes from Burns et al.'s (2012) study, which found that a measure of task switching from an executive function battery was a significant predictor of regret on a task similar to our boxes task.

Burns et al. (2012) measured task switching using a typical set-switching task in which participants had to flexibly change which rule they used to guide a button-press. Whether children experienced regret on the boxes task was selectively related to their performance on trials in which they had to switch rule (in contrast to nonswitch trials). In our study, we were also interested in children's switching behavior, but in a very different sense: We were interested in what we have termed *adaptive choice switching*. That is, we were interested in whether children would appropriately change their choice when faced with the same choice again on a second day. Although adaptive choice switching is very different to the sort of task switching measured by Burns et al., putting our findings together with theirs points to an important way in which the development of emotional and cognitive control processes may mutually interact to lead to age-related changes in decision making. Burns et al.'s findings suggest that a sufficient degree of cognitive flexibility is necessary in order to spontaneously compare and evaluate actual and counterfactual outcomes and thus experience regret; our findings suggest that once children are capable of experiencing regret, this emotional development in turn then allows them to change their behavior adaptively. In other words, although cognitive flexibility may be (at least) part of what is necessary developmentally for experiencing regret, once this emotional ability is intact it feeds into decision-making processes to allow children to adapt and change their behavior appropriately.

If this suggestion is correct, it suggests a quite specific route by which changes in cognitive flexibility can lead to more adaptive and flexible behavior: by supporting the developmental emergence of regret. It is widely accepted that the development of executive processes will have an impact on children's behavior (e.g., Diamond & Lee, 2011; Munakata, Synder, & Chatham, 2012; Stuss, 1992), but the general idea is typically that improvements in these cognitive processes directly impact on children's ability to regulate their behavior appropriately. Here, we are suggesting that they may also do so indirectly: by underpinning the emergence of the functional emotion of regret, which itself then serves to help children adjust their behavior in adaptive ways. This suggestion fits with the general thrust of Baumeister et al.'s (2007) claim that emotions such as regret should be seen as the input to a flexible feedback system that plays a key role behavioral regulation.

It also coheres well with accounts of regret and decision making that have emerged from the neuropsychological literature. Indeed, the functioning of orbitofrontal cortex (OFC) has been described in exactly these sorts of terms, i.e., as integrating cognitive and emotional processes in this way to guide decision making (Camille et al., 2004; Coricelli et al., 2005), and OFC is known to mature over a protracted developmental period (see Happaney, Zelazo, & Stuss, 2004, for review). Coricelli et al. (2005) argue that the experience of regret results in a change in the emotional values associated with choices, biasing against choices that led to a regrettable outcome and triggering an assessment of how behavior needs to change. They argue that the OFC is involved in the evaluative comparison between both actual and counterfactual outcomes, which they view as recruiting declarative cognitive processes, and also in appropriately modifying behavior based on the outcome of this comparison.

Our findings provide new evidence of a role for emotional alongside cognitive development in the development of decision making, and suggest a way to begin to fill the gap identified by others concerning the part played by affective processes in children's decision making (Jacobs & Klaczynski, 2002; Schlottmann & Wilkening, 2011). Much recent research on adult and adolescent decision making has emphasized the role of emotional processes, in part because cognitive measures are not necessarily good predictors of optimal decision making (Toplak, Sorge, Benoit, West, & Stanovich, 2010), and in part because developmental differences between adult and adolescent decision making seem to vary depending on the emotional significance of the task (Figner, Mackinlay, Wilkening, & Weber, 2009; van Duijvenvoorde, Jansen, Visser, & Huizenga, 2010). Our findings suggest a quite specific way in which emotional processes may be important earlier in development: that once children become capable of experiencing regret, their decision making may also improve. This emphasis on regret provides a novel way of thinking about why some young children may be better decision makers than others, or why certain types of decision making may change as children get older.

In summary, we have demonstrated a strong association between experienced regret and adaptive choice switching, and initial evidence that experienced regret can influence decision making independently of the ability to anticipate regret. The exact mechanisms involved are not yet clear, but we have suggested that previously experienced regret may cause decision makers to spontaneously recall the outcomes associated with decision options, thus making them more likely to choose differently when they are faced with the same decision again. We hope that ours are the first of many experiments to investigate the relation between the experience of regret and subsequent decision making in young children. Putting our findings together with those from other developmental studies (Guttentag & Ferrell, 2008; O'Connor et al., 2012; Weisberg & Beck, 2010), a distinct developmental picture of regret is beginning to emerge that has implications for future studies of regret and choice in both children and adults. One important developmental question is whether individual differences in children's ability to experience or anticipate regret are associated with the quality of their decision making more generally. There is much concern about decision quality in children and adolescents (for a review, see Boyer, 2006) and perhaps examining the role of regret will provide a way to predict, and potentially improve, decision quality in young decision makers.
